# Association between Plasma Leptin and Estrogen in Female Patients of Amnestic Mild Cognitive Impairment

**DOI:** 10.1155/2015/450237

**Published:** 2015-11-29

**Authors:** Yi Xing, Jianghong Liu, Jingdong Xu, Linlin Yin, Lin Wang, Junjie Li, Zhipeng Yu, Fangyu Li, Ran Gao, Jianping Jia

**Affiliations:** ^1^Department of Neurology, Xuan Wu Hospital, Capital Medical University, 45 Changchun Street, Beijing 100053, China; ^2^Department of Physiology, School of Basic Medical Sciences, Capital Medical University, 10 Xitoutiao, Youanmen, Fengtai District, Beijing 100069, China; ^3^Department of Pharmacology, Xuan Wu Hospital, Capital Medical University, 45 Changchun Street, Beijing 100053, China; ^4^Beijing Geriatric Medical Research Center, Key Laboratory for Neurodegenerative Disease of Ministry of Education, 45 Changchun Street, Beijing 100053, China

## Abstract

Increasing evidences suggested the association between leptin and cognitive functions. Estrogen is an important factor that regulates the production and metabolism of leptin. However, little is known about the relationship between leptin and estrogen in mild cognitive impairment (MCI). Plasma levels of leptin, total estradiol, and *β*-amyloid protein (A*β*) were measured in a total of 23 female amnestic MCI (aMCI) patients and 19 female cognitively normal controls. This study showed that female aMCI patients had lower plasma levels of leptin and higher levels of estradiol compared to female normal controls. Leptin and estradiol levels were not correlated with cognitive performances or plasma A*β* levels in either aMCI patients or normal controls. There was a significant negative correlation between leptin and estrogen in female aMCI patients (*r* = −0.633, *p* = 0.002) but not in female normal controls. The potential mechanisms of this disease-stage-specific association between leptin and estrogen need further investigations.

## 1. Introduction

Alzheimer's disease (AD) is the major cause of senile dementia. The deposition of extracellular *β* amyloid peptide (A*β*) and the presence of neurofibrillary tangles formed by hyperphosphorylated tau protein are the neuropathological hallmarks of AD. It has been well-known that AD pathological changes begin several years before the onset of dementia. Mild cognitive impairment (MCI) is the symptomatic predementia stage on the continuum of cognitive decline [1]. Considering that neurons have been irreversibly damaged in the dementia stage, the MCI stage might be more valuable for early detection and prevention.

Previous studies have shown that plasma leptin levels were significantly lower in MCI patients than those in normal controls [2]. The longitudinal studies also demonstrated that higher leptin levels were associated with decreased risk of cognitive decline [3]. The laboratory studies identified that the leptin signaling pathways were linked to the pathophysiology of AD. In AD hippocampus, leptin signaling was decreased and its localization was shifted to reactive astrocytes instead of neurons [4]. Accumulating in vivo and in vitro studies have suggested that leptin is associated with decreased A*β* production and tau phosphorylation [5].

The hormone leptin is secreted predominantly by adipocytes and involved in regulating appetite, energy balance, and neuroendocrine. Estrogen is one of the important factors that regulate the metabolism, accumulation, and distribution of adipose tissue and exerts major effects on leptin production. In cell models, estrogen enhanced leptin gene expression [6], and in ovariectomized rats, supplement of estrogen increased leptin concentration [7]. Furthermore, estrogen plays important roles in pathogenic mechanisms of AD. Numerous studies have demonstrated the neuroprotective effects of estrogen. Estrogen can inhibit the formation of A*β* plaques and neurofibrillary tangles [8]. Thus, all these evidences showed that there is a close relationship between leptin and estrogen and both of them influence the pathophysiological changes of AD. However, in MCI stage, the study of association between leptin and estrogen is still lacking.

In this study, we selected female amnestic MCI (aMCI) patients as our study subjects. The longitudinal clinical-pathological studies have identified that aMCI patients are on a pathway toward AD, and 75–80% of aMCI patients finally convert to AD [9]. We investigated the changes of leptin and estrogen levels in female aMCI patients compared to those in normal controls and the relationship between leptin and estrogen in aMCI. We were also interested in the influences of leptin and estrogen on memory function and plasma A*β* levels.

## 2. Methods

### 2.1. Subjects

The aMCI patients and cognitively normal controls were recruited from the memory clinic of Xuan Wu Hospital from January 2012 to March 2014. For all of our patients, aMCI was a consensus diagnosis made by at least two experienced neurologists. These neurologists made the diagnosis strictly according to the published criteria [10, 11]: cognitive concern by subject, informant, nurse, or physician; impairment in 1 or more of the 4 cognitive domains; essentially normal functional activities; and absence of dementia. Subjects with MCI were classified as aMCI if the memory domain was impaired.

All the controls had normal cognitive functions, assessed by the Mini-Mental State Examination (MMSE), Montreal Cognitive Assessment (MoCA), and the Clinical Dementia Rating (CDR) scale, and none of them had previous history of neurological or psychiatric disorders.

Subjects with acute illnesses or taking exogenous hormones currently or previously were excluded. All the subjects were postmenopausal in this study. Written informed consents were obtained from all participants or their relatives before the study. This study was approved by the Institutional Review Board of Xuan Wu Hospital.

### 2.2. Assessments

Both cases and controls were asked to provide information on age, years of formal education, height, and weight. The body mass index (BMI) was calculated as weight (kilograms) divided by squared height (meters). The MMSE and MoCA were used to assess global cognitive ability [12, 13]. The CDR was used to examine the different cognitive domains [14]. We used the World Health Organization-University of California-Los Angeles Auditory Verbal Learning Test (WHO-UCLA AVLT) to measure verbal memory [15]. In this test, the subjects were asked to recall a 15-word list right after the presentation. This kind of immediate recall was repeated 3 times (maximum score = 45). Thirty minutes later, the subjects performed the long delay free recall (maximum score = 15) and long delay recognition (maximum score = 15).

### 2.3. Plasma Examinations

Plasma samples were obtained in the morning following an overnight fast. The samples were kept at −80°C until being analyzed. Plasma leptin levels were measured by a commercial enzyme-linked immunosorbent assay (ELISA) kit (R and D Systems, Inc., Minneapolis, MN, USA). The lower limit of detection was 7.8 pg/mL. The interassay precision was 3.5–5.4%, and the intra-assay precision was 3.0–3.2%. The plasma levels of estradiol were detected by an electrochemiluminescence immunoassay (cobas e 601, Roche Diagnostics GmbH, Mannheim, Germany) [16]. The detection limit of estradiol was 18.4 pmol/L. The intra-assay coefficient of variation (CV) at 130 pmol/L was 3.3%, and interassay CV at 120 pmol/L was 4.7%. The plasma levels of A*β*_42_ and A*β*_40_ were determined by ELISA kit (Invitrogen, USA) [17]. The intra-assay and interassay CVs for A*β*_42_ were 3.1% and 4.0%, respectively. For A*β*_40_, the intra-assay and interassay CVs were 2.4% and 3.6%, respectively.

### 2.4. Statistical Analysis

Normality of distribution was examined using Kolmogorov-Smirnov test. We used independent samples *t*-tests to explore the characteristics of our subjects, including age, education, BMI, and cognitive scores. However, the levels of A*β*_42_ and A*β*_40_ were skewed distribution, so they were analyzed with a nonparametric Mann-Whitney *U* test. For comparisons of leptin and estrogen levels between aMCI group and normal controls, linear regression models were constructed. The levels of leptin and estrogen were added to regression models as dependent variables, respectively; disease status (aMCI or normal control) was independent variable, and age and BMI were adjusted. We analyzed the correlations using Pearson or Spearmen tests when involving A*β*_42_ and A*β*_40_ levels. For the correlations between leptin and estradiol and cognitive scores, the partial correlation analyses were used to control confounded factors, including age, education, and BMI. A *p* value <0.05 was regarded as statistically significant.

## 3. Results

### 3.1. Subjects' Characteristics

A total of 23 female aMCI patients and 19 female cognitively normal controls were included. The characteristics of our subjects were presented in Table 1. Age, education, and BMI were matched between these two groups. All the aMCI patients had a CDR score of 0.5, and all the controls had a CDR score of 0. The MMSE and MoCA scores were significantly lower in aMCI patients than those in normal controls. The aMCI patients performed significantly worse in memory tests compared to the controls. There were no group differences in the plasma levels of A*β*_42_, A*β*_40_, or A*β*_40_/A*β*_42_.

### 3.2. Comparisons of Plasma Leptin and Estradiol Levels between aMCI Patients and Normal Controls

All aMCI patients had the levels of estradiol higher than the detection limit. Two normal controls had undetectable plasma estradiol levels, which were regarded as the lower detection limit (18.4 pmol/L). The comparisons of plasma leptin and estradiol levels between groups were shown in Table 2. The aMCI patients had significantly lower plasma leptin levels than normal controls; however, their plasma estradiol levels were significantly higher than those in normal controls. After adjusting for age and BMI, the results were still unchanged.

### 3.3. Correlations between Leptin, Estrogen, Cognitive Functions, and Plasma A*β* Levels

**Figure 1:**
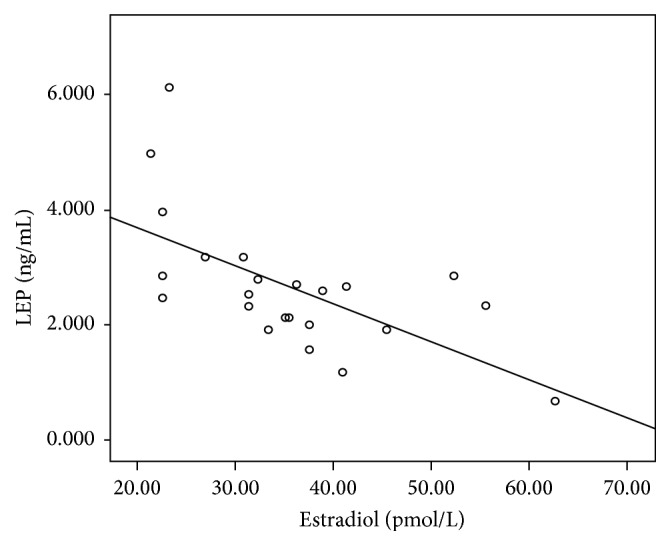
The scatter plot for the correlation between estradiol and leptin in aMCI.

The correlation analyses were conducted in aMCI patients and normal controls, respectively. In normal controls, there was no correlation between leptin and estradiol levels. Neither leptin nor estradiol was correlated with any cognitive tests, and further adjustment for age, education, and BMI did not change the results. Leptin and estradiol levels were not correlated with plasma A*β* levels (including A*β*_42_, A*β*_40_, and A*β*_40_/A*β*_42_) in normal controls. In aMCI patients, similarly, the levels of leptin and estradiol were not correlated with the scores of cognitive examinations or plasma A*β* levels. However, there was a significantly negative correlation between leptin and estradiol levels in aMCI patients (*r* = −0.621, *p* = 0.002). This correlation was also shown in the scatter plot (Figure 1).

## 4. Discussion

An increasing number of evidences suggested the association between leptin and AD, and numerous laboratory studies have demonstrated the extensive roles that estrogen plays in the pathogenic mechanisms of AD. Interestingly, there is a close relationship between estrogen and leptin; estrogen is one of the important regulating factors in the metabolism of leptin [18, 19]. Some previous researches have studied the links between leptin and estrogen in normal females; however, to our knowledge, our study is the first one that investigated the association between leptin and estrogen in aMCI patients. The results of this study showed that (1) female aMCI patients had lower levels of leptin and higher levels of estradiol compared to female normal controls, (2) there was a significant negative correlation between leptin and estradiol levels in female aMCI patients, but leptin and estradiol levels were not correlated in cognitively normal women, and (3) leptin and estradiol levels were not correlated with the scores of cognitive tests or plasma A*β* levels in either aMCI patients or normal controls.

In previous cross-sectional studies, plasma leptin levels were decreased in MCI patients [2]. In longitudinal studies, higher leptin levels were associated with a lower risk of AD and cognitive decline [3, 20, 21]. Our result of lower leptin levels in aMCI patients was consistent with these studies. However, some other studies showed that the plasma levels of leptin were not altered in MCI patients and not associated with the risk of cognitive decline [22–24]. Based on the controversial results, one study identified the influences of race and gender, and the result suggested that leptin levels were negatively associated with the scores of cognitive tests in black men but showed an opposite effect in white men; more interesting, for women, there was no relationship between leptin levels and cognitive performances in this previous study [25]. In our study, all subjects were female and no correlation was found between leptin and cognitive performances. Thus, the influences of gender and race should be considered in further studies on leptin and cognition, and our results need confirmation in larger Asian samples.

Although the exact change of leptin in cognitive impairment population was still inconsistent, many laboratory studies had suggested the neuroprotective effects of leptin against AD [26]. Multiple studies demonstrated that leptin reduced A*β* production and downregulated the activity and expression levels of BACE1 [27]. The plasma leptin was strongly negatively associated with A*β* levels in the mouse brain [28]. Thus, in our study, we investigated the correlation between leptin and plasma A*β* levels (including A*β*_42_, A*β*_40_, and A*β*_40_/A*β*_42_). However, no correlation was found in either aMCI patients or normal controls. Similarly, using CSF, the previous study also showed no correlation between CSF levels of leptin and A*β*_42_ [4].

The most interesting finding of our study was the higher estradiol levels in aMCI patients and the negative correlation, which was not found in normal controls, between plasma leptin and estradiol levels in aMCI patients. Although estrogen is usually regarded as a neuroprotective factor, the effects of estrogen on AD are still ambiguous in population studies. Some studies reported comparable or even higher levels of estrogen in patients with AD [29, 30]. Consistent with previous studies [31–33], our study did not find the correlation between leptin and estradiol in cognitively normal postmenopausal women. However, in female aMCI patients, the leptin levels were significantly negatively correlated with estradiol. The “healthy cell bias of estrogen action” hypothesis may partially explain these inconsistent results [34]. This hypothesis suggests that estrogen has beneficial effects on neurological function if neurons are intact; however, oppositely, if neurological health has already been compromised, estrogen may exacerbate neurological impairment.

There are some limitations in our study. First, as a preliminary study, our sample size is relatively small. Since there was no previous study that investigated the relationship between leptin and estrogen in aMCI patients, our result needs confirmation in larger samples. For the aim of our study, the study samples were females only and our results are probably not suitable for men, and considering the possible influences of race on the results, our results may be more valuable for Asian population. Furthermore, the potential confounded factors were controlled when we analyzed the data; however, the influences of unknown or unmeasured factors cannot be excluded. Last, the cross-sectional study design precludes the explanation of causal relationship.

## 5. Conclusions

In conclusion, our study is valuable for further observation and intervention studies on leptin and estrogen in MCI patients. As a preliminary study, our results suggested that there were opposite changes in plasma leptin and estrogen levels in female aMCI patients. Interestingly, the significant negative correlation between leptin and estrogen existed in female aMCI patients but not in female normal controls. Our study suggested that there may be a disease-stage-specific association between leptin and estrogen, and the potential mechanisms deserve further investigations.

## Figures and Tables

**Table 1:** The characteristics of our subjects.

	aMCI, *n* = 23	Control, *n* = 19	*p*
Age	67.2 (6.9)	68.1 (7.3)	0.707
Education (year)	9.4 (3.1)	11.3 (3.4)	0.073
BMI	23.5 (1.5)	24.7 (3.1)	0.110
Cognitive tests			
MMSE	24.6 (2.9)	27.7 (1.7)	<0.001
MoCA	20.4 (3.2)	25.8 (2.5)	<0.001
Immediate verbal recall	15.1 (3.2)	24.7 (4.4)	<0.001
Delay free recall	3.6 (1.5)	8.7 (2.3)	<0.001
Delay recognition	6.9 (2.2)	12.1 (1.6)	<0.001
^*∗*^Plasma A*β*_42_ (pg/mL)	24.6 (20.8, 27.3)	22.2 (19.8, 29.7)	0.695
^*∗*^Plasma A*β*_40_ (pg/mL)	108.3 (89.2, 136.7)	100.7 (90.9, 148.6)	0.771
Plasma A*β*_40_/A*β*_42_	4.5 (0.6)	4.8 (0.9)	0.258

Values are means (SDs).

^*∗*^Values are medians (interquartile ranges).

**Table 2:** Comparisons of plasma leptin and estradiol levels between aMCI patients and normal controls.

	aMCI, *n* = 23	Control, *n* = 19	*β*	*p*
Plasma leptin (mean ± SD, ng/mL)	2.6 (1.1)	6.7 (4.2)		
Unadjusted			1.165	<0.001
Adjusted			1.339	<0.001
Plasma estradiol (mean ± SD, pmol/L)				
Unadjusted	35.5 (10.8)	28.4 (7.1)	−0.093	0.029
Adjusted			−0.118	0.019

Adjusted for age and BMI.
